# The Role of Zinc in the Development of Vascular Dementia and Parkinson’s Disease and the Potential of Carnosine as Their Therapeutic Agent

**DOI:** 10.3390/biomedicines12061296

**Published:** 2024-06-11

**Authors:** Dai Mizuno, Masahiro Kawahara, Keiko Konoha-Mizuno, Ryoji Hama, Terumasa Ogawara

**Affiliations:** 1 Department of Forensic Medicine, Faculty of Medicine, Yamagata University, 2-2-2 Iida-Nishi, Yamagata-shi 990-9585, Yamagata, Japan; k_mizuno@med.id.yamagata-u.ac.jp (K.K.-M.); hama-ryoji@med.id.yamagata-u.ac.jp (R.H.); t.ogawara@med.id.yamagata-u.ac.jp (T.O.); 2 Research Institute of Pharmaceutical Sciences, Faculty of Pharmacy, Musashino University, 1-1-20 Shin-machi, Nishitokyo-shi 202-8585, Tokyo, Japan; makawa@musashino-u.ac.jp

**Keywords:** apoptosis, carnosine, endoplasmic reticulum stress, Parkinson’s disease, synapse, vascular dementia, zinc

## Abstract

Synaptic zinc ions (Zn^2+^) play an important role in the development of vascular dementia (VD) and Parkinson’s disease (PD). In this article, we reviewed the current comprehension of the Zn^2+^-induced neurotoxicity that leads to the pathogenesis of these neuronal diseases. Zn^2+^-induced neurotoxicity was investigated by using immortalised hypothalamic neurons (GT1-7 cells). This cell line is useful for the development of a rapid and convenient screening system for investigating Zn^2+^-induced neurotoxicity. GT1-7 cells were also used to search for substances that prevent Zn^2+^-induced neurotoxicity. Among the tested substances was a protective substance in the extract of Japanese eel (*Anguilla japonica*), and we determined its structure to be like carnosine (β-alanylhistidine). Carnosine may be a therapeutic drug for VD and PD. Furthermore, we reviewed the molecular mechanisms that involve the role of carnosine as an endogenous protector and its protective effect against Zn^2+^-induced cytotoxicity and discussed the prospects for the future therapeutic applications of this dipeptide for neurodegenerative diseases and dementia.

## 1. Introduction

Zinc (Zn) plays an important role in a variety of physiological functions, including cell mitosis, the immune system, and protein and nucleic acid synthesis, and it acts as a cofactor for over 300 enzymes or metalloproteins, contributing to normal brain function [1]. Nevertheless, Zn is widely known to play an important role in the development of post-ischaemic neurodegeneration and vascular dementia (VD) [2]. Recently, it has been reported that Zn is involved in the mechanism of Parkinson’s disease (PD) pathogenesis [3].

Senile dementia is characterised by severe memory loss and an inability to form new memories in the elderly, and its prevalence increases with age. A fact sheet published by the World Health Organization in 2023 states that there are 55 million people with dementia worldwide, an increase of nearly 10 million each year [4]. Dementia is a serious social problem in rapidly ageing societies. Senile dementia is classified into Alzheimer’s disease (AD), VD, and dementia with Lewy bodies (DLB). VD is considered the second most common type of dementia, making up approximately 20–40% of senile dementias [5]. Both AD and DLB are characterised by abnormally accumulated protein deposits (β-amyloid protein (AβP) in AD and α-synuclein in DLB) in the brain, whereas VD is a degenerative cerebrovascular disease that comes with a series of strokes or ischaemia [6,7,8]. PD is a progressive neurodegenerative disease that presents with motor deficits, such as resting tremors, muscle rigidity, akinesia, and impaired postural reflexes and develops in people over the age of 60 years at a rate of approximately 1 in 100 [9,10]. PD is caused by a loss of dopamine neurons in the substantia nigra and the promotion of inflammatory responses by microglia at the lesion site [11,12]. PD is a multifactorial disease involving genetic and environmental factors for which age is the greatest risk factor [13,14,15]. Environmental factors include exposure to pesticides, herbicides, and heavy metals; smoking; and caffeine consumption [12].

Previously, we revealed the influence of calcium (Ca) dyshomeostasis, disruptions to energy production pathways, and endoplasmic reticulum (ER) stress pathways on the molecular mechanisms of Zn^2+^-induced neurotoxicity [16,17,18]. Moreover, we found that copper (Cu) activated oxidative stress, the ER stress response, and mitochondrial injury and enhanced Zn^2+^-induced GT1-7 cell death during examinations regarding the effects of various metal ions on Zn^2+^-induced neurotoxicity [19,20,21]. The activation of the Zn^2+^-dependent stress-activated protein kinase/c-Jun amino-terminal kinase (SAPK/JNK) pathway is important for neuronal cell death, and Cu^2+^-induced oxidative stress triggers this activation [22]. Based on these findings, we hypothesised that these molecular pathways are related to Zn^2+^-induced neurotoxicity.

**Figure 1:**
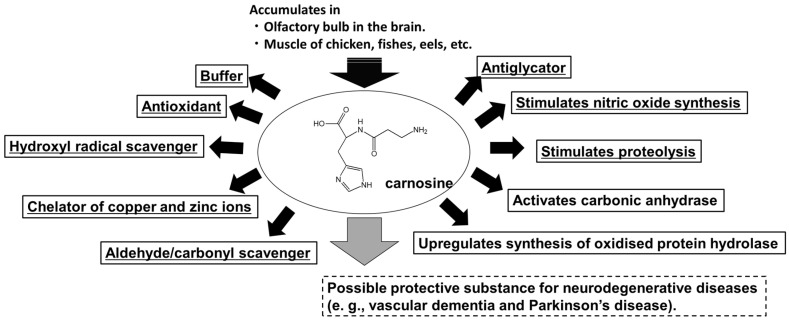
Structures and roles of carnosine.

Substances that reduce Zn^2+^-induced neurotoxicity may be agents for the treatment or prevention of neurological diseases such as VD and PD [23,24]. During the search for a protective substance derived from extracts of various agricultural products using a screening system for protective substances using GT1-7 cells, we found that carnosine (β-alanylhistidine) exhibited a marked inhibitory effect on Zn^2+^-induced neurotoxicity and proposed that it is a candidate drug for the treatment of VD [25]. Carnosine is an endogenous dipeptide with various useful properties such as antioxidation, anti-glycation, and anti-crosslinking (Figure 1) [26,27]. Carnosine accumulates abundantly in the skeletal muscle and olfactory bulb. The olfactory bulb is responsible for transmitting external information and substances, presumably protecting neurons from external toxins, and acting as an endogenous protector against damage and aging. Carnosine levels change during development and decrease with age [28]. This may be associated with an increased risk of neuropathy with ageing. Carnosine supplementation in older adults may reduce this risk. In this article, based on our own and other previous studies, we focus on the molecular mechanisms of Zn^2+^-induced neurotoxicity and the properties of carnosine against this disease and discuss potential therapeutic agents for VD and PD.

## 2. Zn Neurotoxicity

### 2.1. Usefulness of GT1-7 Cells in the Study of Zn^2+^-Induced Neurotoxicity

**Figure 2:**
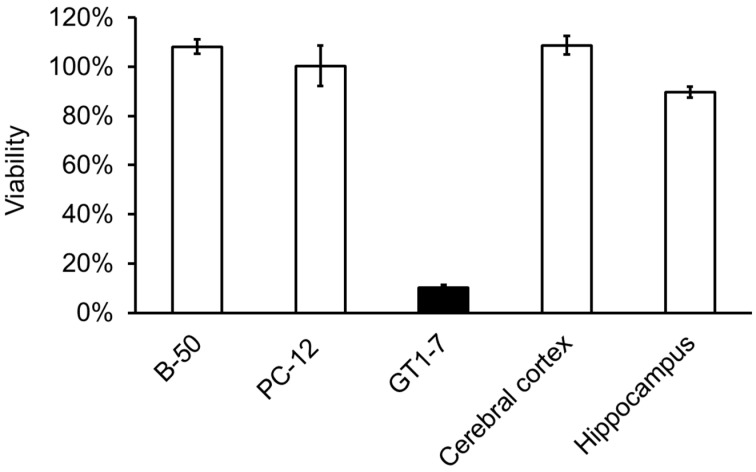
A comparison of Zn^2+^ cytotoxicity in GT1-7 cells with that of other neuronal cells. Various neuronal cells were exposed to Zn^2+^, and cytotoxicities were evaluated using their viabilities. Cultured neuronal cells, including GT1-7, PC-12, and B-50 cells (a neuroblastoma cell line), and primary cultured neurons from the rat cerebral cortex and hippocampus, were administered 50 μM of ZnCl_2_. The cell viability was analysed using the WST-1 method 24 h after administration. This figure was created using previously reported data [16,17].

It has been recognized that abnormalities in Zn homeostasis (e.g., excess or deficiency) are involved in neurological diseases such as VD [2]. Understanding the molecular mechanism of neuronal cell death induced by Zn^2+^, which accounts for a large amount of Zn in the brain, is important for elucidating the pathogenesis of VD and developing therapeutic agents. We have shown that Zn^2+^ causes cell death in immortalised hypothalamic neurone GT1-7 cells [16,17]. In our studies, GT1-7 cells were more vulnerable to Zn^2+^ cytotoxicity than other neuronal cells, including primary cultures of rat cortical and hippocampal neurons, PC-12 cells, and B-50 cells (Figure 2). GT1-7 cells possess neuronal characteristics such as neurite extension; the secretion of gonadotropin-releasing hormone (GnRH); and the expression of neurone-specific proteins and receptors, including microtubule-associated protein 2, tau protein, neurofilament, synaptophysin, GABA_A_ receptors, dopamine receptors, and L-type Ca^2+^ channels [29]. Zn^2+^ is released along with glutamate upon glutamatergic neuronal excitation [30]. As glutamate also causes neurotoxicity, it is difficult to exclude the effects of glutamate from Zn^2+^ neurotoxicity in cells with glutamate receptors. By contrast, GT1-7 cells exhibit little cytotoxicity to glutamate since the expression of ionotropic glutamate receptors in this cell line is lacking or at possess low levels [31]. These characteristics make this cell line a useful model for investigating Zn^2+^-induced neurotoxicity.

### 2.2. Molecular Mechanism of Zn^2+^-Induced GT1-7 Cytotoxicity

#### 2.2.1. Disruption of Calcium Homeostasis

Zn^2+^-treated GT1-7 cells are positive for transferase-mediated biotinylated UTP nick-end labelling, indicating that Zn^2+^ induces apoptosis in GT1-7 cells [16,17]. Comprehensive Screening using DNA microarray and analyses using real-time PCR (RT-PCR) have revealed that the administration of Zn^2+^ to GT1-7 cells induces the expression of various genes, including in metal-related genes ((Zn transporter 1 [*ZnT-1*], metallothionein [*MT*]*1*), and *MT2*), ER-stress-related genes, and Ca^2+^ signalling transmission-related genes [19,32,33]. The administration of Zn^2+^ to GT1-7 cells also increased intracellular Ca^2+^ levels ([Ca^2+^]_i_). Apoptosis is inhibited by pyruvate, citrate, and Ca^2+^ channel antagonists (nifedipine, conotoxin, and Al^3+^) [16,17,18]. The changes in [Ca^2+^]_i_ after exposure to Zn^2+^ were observed by using a high-resolution multisite video imaging system with Fura-2 as a fluorescent probe for cytosolic Ca^2+^ [17,34]. This revealed that pretreatment with Al^3+^, which acts as various kinds of Ca^2+^ channel blockers [35], suppressed the elevation of [Ca^2+^]_i_ levels in Zn^2+^-treated GT1-7 cells. Although Al was neurotoxic, Al^3+^ did not affect GT1-7 cellular viability under these experimental conditions [18] since it has difficulty entering cells without a membrane-permeable chelator [36]. Zn^2+^-induced GT1-7 cell death may be attenuated by Al^3+^, which suppresses the elevation of the [Ca^2+^]_i_ level. Therefore, Ca^2+^ homeostasis is likely to be involved in Zn^2+^-induced GT1-7 cytotoxicity. 

#### 2.2.2. Energy Deficiency and Mitochondrial Glycolysis Inhibition

We previously showed that the energy substrates pyruvate and citrate salts inhibit Cu^2+^- and Zn^2+^-induced GT1-7 cell death [21]. The coexistence of pyruvate and citrate did not affect [Ca^2+^]_i_, intracellular Zn^2+^ levels ([Zn^2+^]_i_), or *MT* mRNA levels. Therefore, it is unlikely that pyruvate and citrate attenuated Cu/Zn-induced neurotoxicity by chelating Cu^2+^ and Zn^2+^ [21]. It has been reported that nicotinamide adenine dinucleotide (NAD^+^) and ATP levels are decreased by Zn exposure in cultured cortical neurons, and NAD^+^ levels are restored through treatment with pyruvate [37,38]. Pyruvate administration also attenuates post-ischaemic neuronal cell death in vivo [39]. Imaging studies using Zn^2+^-sensitive fluorescent dyes and mitochondrial markers have revealed that Zn^2+^ is localised within mitochondria [40]. It has been reported that Zn^2+^ can inhibit various mitochondrial enzymes and the intracellular trafficking of mitochondria [41]. Taken together, these results suggest that energy deficiency and the inhibition of mitochondrial glycolysis are involved in Zn^2+^ neurotoxicity.

#### 2.2.3. ER Stress Pathway Involvement in Zn^2+^-Induced Cytotoxicity

DNA microarrays have revealed that Zn^2+^ markedly increases several gene expressions, including ER-stress-related genes (CCAAT enhancer-binding protein homologous protein (*CHOP*); growth arrest and DNA damage-induced gene 34 (*GADD34*)) and a Ca^2+^-related gene (*Arc*) [32,33]. A decreased Ca^2+^ level in the ER is thought to cause ER stress because it leads to decreased function in molecular chaperones and enzymes that bind Ca^2+^ [42]. Since the ER acts as an intracellular Ca^2+^ reservoir that contains much more Ca^2+^ than cytosol and is involved in the regulation of [Ca^2+^]_I_, an increase in the [Ca^2+^]_i_ in Zn^2+^-treated GT1-7 cells can be associated with a decrease in the Ca^2+^ level in the ER. The increase in [Ca^2+^]_i_ in GT1-7 cells induced by Zn^2+^ administration appears to be closely related to the upregulation of these ER-stress-related factors. ER stress, which occurs owing to the accumulation of misfolded and unfolded proteins, is implicated in various neurological diseases such as AD, prion disease, and cerebral ischaemia [43]. Three signalling proteins, called ER stress sensors—inositol-requiring enzyme 1α (IRE1α), protein kinase R-like ER kinase (PERK), and activating transcription factor (ATF) 6—are activated by sensing ER stress [44]. IRE1α, PERK, and ATF6 activate diverse signalling pathways. The phosphorylation of the α-subunit of eukaryotic translation initiation factor 2α regulates ATF4 translation via PERK. ATF4 is a transcription factor that promotes *CHOP* and *GADD34*. RT-PCR has confirmed that Zn^2+^ administration to GT1-7 cells induces the expression of *Arc*, *CHOP*, *GADD34*, *ATF4*, and metal-related genes (*ZnT-1*, *MT1*, and *MT2*), whereas other ER-stress-related genes, including ER degradation-enhancing α-mannosidase-like protein (*EDEM*), glucose-regulated protein 94 (*GRP94*), protein disulfide isomerase (*PDI*), immunoglobulin binding protein (*Bip*), and spliced X-box binding protein-1 (*sXBP1*), show no significant changes on account of Zn^2+^ administration. Furthermore, dantrolene, an inhibitor of ER stress, attenuates Zn^2+^-induced GT1-7 cytotoxicity [27]. These results strongly suggest that PERK-related pathways are involved in Zn^2+^-induced ER stress.

#### 2.2.4. Cu Enhances Zinc-Induced GT1-7 Cell Death

**Figure 3:**
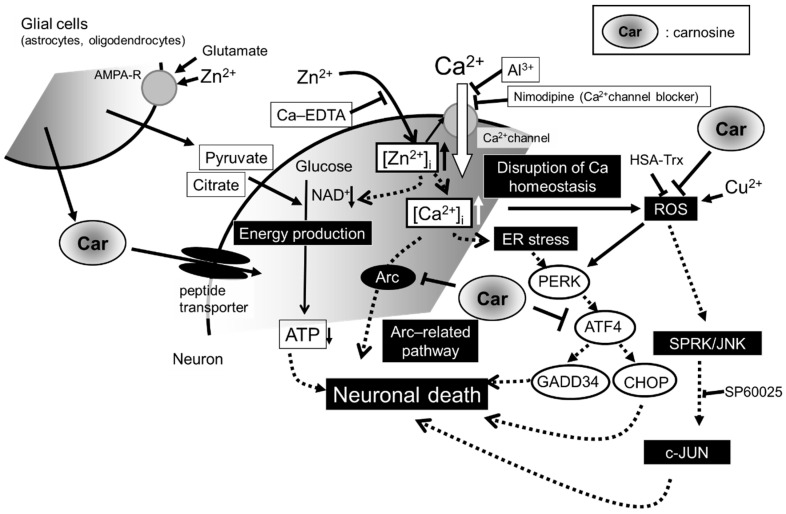
Hypothetical illustration of the mechanism involved in the protective effect of carnosine in preventing zinc-induced neuronal death. Zn^2+^ is secreted from presynaptic vesicles into the synaptic cleft during ischaemia. Excess Zn^2+^ translocates into the cell and can cause a disruption in Ca^2+^ homeostasis; mitochondrial energy failure; endoplasmic reticulum (ER); oxidative stress; and, consequently, apoptotic neuronal death. The co-exposure of Cu^2+^, which is secreted with Zn^2+^ during ischaemia, potentiates these effects. Zn^2+^ chelators (Ca-EDTA), Ca^2+^ channel blockers (Al^3+^ and nimodipine), energy substrates (pyruvate and citrate), the SAPK/JNK signalling pathway inhibitor (SP600125), and antioxidants (HSA-Trx) inhibit these pathways. Carnosine (Car) inhibits the ER-stress-related, Ca^2+^-related gene (*Arc*)-related apoptotic, and reactive oxygen species (ROS) pathways. Carnosine is secreted from glial cells in response to glutamate and Zn^2+^ stimulation and protects neurons from Zn^2+^ neurotoxicity. The solid and dotted lines in the figure represent the movement of substances and the pathways of cell death, respectively.

In addition to Zn, trace elements such as iron (Fe), Cu, and manganese (Mn) are distributed at different levels in various areas of the brain and maintain its normal structure and function [45,46]. Of these metals, we showed that the presence of Cu^2+^ markedly exacerbated Zn^2+^-induced GT1-7 cytotoxicity [19]. Cu^2+^ alone did not affect the gene expression levels of *Arc*, *CHOP*, or *GADD34* but significantly enhanced the induction of these factors by Zn^2+^. Furthermore, Western blotting showed that the co-administration of Zn^2+^ and Cu^2+^ significantly increased the amount of CHOP protein. CHOP is involved in the initiation of the apoptotic cascade [47] and GADD34 activation, which reportedly increases after traumatic brain injury [48]. Furthermore, it has been reported that the antioxidant thioredoxin-conjugated human serum albumin (HSA-Trx) attenuates Cu^2+^- and Zn^2+^-induced neuronal cell death [20]. Zn exists only as Zn^2+^, whereas Cu is a redox-active metal that exists as oxidised Cu^2+^ and reduces Cu^+^. Cu^2+^ administration induces reactive oxygen species (ROS) generation in GT1-7 cells, whereas Zn^2+^ alone does not induce ROS generation or affect Cu^2+^-induced ROS generation [22]. The involvement of oxidative stress in various neurodegenerative diseases is well known, and ROS that cause oxidative stress adversely affect many signalling pathways, such as the SAPK/JNK-related and ER-related pathways [49,50,51,52]. The co-administration of Cu^2+^ and Zn^2+^ to GT1-7 cells activates SAPK/JNK, phospho-c-Jun, and phospho-ATF2 downstream of the SAPK/JNK pathway. Furthermore, SP600125, an inhibitor of the SAPK/JNK signalling pathway, significantly suppresses Cu^2+^- and Zn^2+^-induced SAPK/JNK signalling pathway activation and neuronal cell death [22]. In addition, the suppression of Cu^2+^ and Zn^2+^ cytotoxicity by HSA-Trx inhibits the SAPK/JNK signalling pathway activation and ROS production [47]. Furthermore, the endogenous selenium (Se)-containing amino acid selenomethionine (Se-Met) induces glutathione peroxidase, blocks ROS production, significantly inhibits CHOP induction, and inhibits Cu^2+^- and Zn^2+^-mediated cytotoxicity [53]. Cu^2+^ triggers ROS production, which may enhance Zn^2+^ cytotoxicity by inducing the SAPK pathway and ER stress [20,53]. These findings indicate that Zn^2+^ may be significantly involved in the ER stress pathway. Figure 3 shows our hypotheses regarding Zn^2+^-induced neuronal death (and the protective effect of carnosine, which will be discussed later). 

### 2.3. Role of Zn in the Development of VD and PD

#### 2.3.1. Zn-Related Neurotoxicity

Based on the aforementioned results, the hypotheses regarding Zn^2+^-related neurotoxicity are as follows (Figure 3). Normally, Zn^2+^ and Cu^2+^ are released into the synaptic cleft upon neuronal excitation and regulate signal transduction [31]. Secreted Zn^2+^ and Cu^2+^ undergo rapid reuptake into presynaptic neurons via the Zn transporter or CTR1, thereby maintaining the level of these ions in the synaptic cleft. However, under conditions such as transient global cerebral ischaemia, which may be associated with VD development, prolonged neuronal excitation occurs in major areas in the brain, and Zn^2+^ and Cu^2+^ are released into the synaptic cleft and translocate to the same neurons in large amounts. Increased [Zn^2+^]_i_ inhibits mitochondrial energy production mechanisms. Pyruvate and citrate, the energy substrates, prevent this process [21]. Zn^2+^ also leads to an increase in [Ca^2+^]_i_ [17,18]. Impaired cellular protein folding due to energy depletion causes the accumulation of defective proteins in the ER. An increase in [Ca^2+^]_i_ induces ROS generation. This potentiates ER stress and/or the SAPK/JNK pathways, leading to apoptotic neuronal cell death [49,50,51,52].

#### 2.3.2. VD and Zn

There is increasing evidence that Zn^2+^-mediated post-ischaemic neuronal cell death is involved in neurodegeneration after stroke or ischaemia [54,55,56,57]. VD is a disease associated with this form of neurodegeneration [8,58]. Under the conditions of transient global ischaemia or stroke, prolonged neuronal excitations in most areas of the brain are due to the blockage of blood flow and concomitant oxygen and glucose deprivation. The excessive release of glutamate from synaptic vesicles into the synaptic cleft follows this abnormal excitation. In the hippocampus or cerebral cortex, the delayed cell death of vulnerable neurons is caused by a constant influx of Ca^2+^, leading to the development of infarcts, cognitive impairment, and VD. Epidemiological studies have reported that approximately 30% of patients with stroke develop symptoms of dementia 3 years later [59]. Under ischaemic conditions, significant amounts of Zn^2+^ (approximately 300 μM) have been reported to be released into the synaptic cleft along with glutamate after membrane depolarisation [60]. Zn^2+^ entry and increased [Zn^2+^]_i_—in other words, ‘Zn translocation’ to postsynaptic neurons—reportedly occur in degenerated hippocampal neurons after ischaemia [61]. This Zn translocation is controlled by VDCC, the *N*-methyl- d-aspartic acid (NMDA)-type glutamate receptor, and Ca^2+^-permeable α-amino-3-hydroxy-5-methyl-4-isoxazolepropionate (AMPA)-type glutamate receptor [2]. The administration of a membrane-impermeable Zn^2+^ chelator (calcium ethylenediaminetetraacetate [Ca-EDTA]) has protected hippocampal neurons and reduced infarct volume after transient global ischaemia in experimental animals [61]. Kitamura et al. revealed an increase in extracellular Zn^2+^ levels in rats with transient middle cerebral artery occlusion using microdialysis [62]. Additionally, Zn^2+^ contributes to increased blood–brain barrier (BBB) permeability following ischaemia [63]. Given these results, it is strongly suspected that Zn plays an important role in delayed neuronal death after the onset of transient global ischaemia, as well as the pathogenesis of VD [56]. The molecular mechanism of Zn-induced neurotoxicity is associated with the ER stress pathway, mitochondrial energy failure, Ca homeostasis disruption, the MAP kinase pathway (SAPK/JNK pathway), and ROS production [64]. Furthermore, the addition of Cu^2+^ produces ROS, which is known to induce ER stress and activate SAPK/JNK pathways, and significantly exacerbates Zn-induced neurotoxicity [19]. Several antioxidants attenuate Cu- and Zn-induced neurotoxicity. Therefore, Cu may play an important role in Zn-induced neurotoxicity by producing ROS, ultimately leading to VD development.

#### 2.3.3. PD and Zn

Zn^2+^-mediated neuronal cell death may be involved in the pathogenesis of PD [3]. Dopaminergic neuronal shedding and microglial activation, which are implicated in the pathogenesis of PD, have been shown in animal models of PD established using 1-methyl-4-phenyl-1,2,3,6-tetrahydropyridine or 6-hydroxydopamine (6-OHDA) [65]. ROS derived from 6-OHDA uptake through dopamine transporters and intraneuronal 6-OHDA autoxidation, extracellular 6-OHDA autoxidation, and microglial activation are known to be molecular species involved in mechanisms responsible for 6-OHDA-induced dopaminergic degeneration [66]. 6-OHDA is easily oxidised and generates several cytotoxic products, such as quinones, H_2_O_2_, 5,6-dihydroxyindole, superoxide anion radicals, hydroxyl radicals, and singlet oxygen [67]. ROS derived from paraquat (PQ), a herbicide that is also taken up by dopamine transporters, leads to glutamate exocytosis via transient receptor potential melastatin 2 cation channel activation in the substantia nigra [68,69]. Signal transduction by extracellular glutamate induces extracellular Zn^2+^ influx via AMPA receptor activation, followed by nigral dopaminergic degeneration via intracellular Zn^2+^ dysregulation. From the intracellular ROS derived from 6-OHDA and PQ, H_2_O_2_ easily passes through cell membranes via aquaporin channels [70]. H_2_O_2_ elevation in the extracellular compartment induces Zn^2+^ release along with glutamate through the excitement of glutamatergic neurone terminals [71]. The released Zn^2+^ may cause Zn translocation similar to that observed in the hippocampus post-ischaemia. Additionally, we showed that treatment with 6-OHDA induces integrated stress-related genes, such as *CHOP*, *GADD34*, and *ATF4* in GT1-7 cells [24]. Increased Zn^2+^ release caused by 6-OHDA may be related to Zn translocation, which induces the release of these factors.

#### 2.3.4. Carnosine Prevents Zn^2+^-Induced Neurotoxicity

Zn translocations are key events in Zn^2+^-induced neurotoxicity. Voltage-gated Ca^2+^ channels, NMDA-type glutamate receptors, and AMPA/kainate-type glutamate receptors (A/K-R) are major pathways for Zn^2+^ entry [72]. Hippocampal neurons have poor permeability to Ca^2+^ and Zn^2+^ since they mostly express AMPA receptors containing GluR2 subunits under normal conditions. However, after ischaemia, there is a sharp decrease in GluR2 expression, and neurons express Ca^2+^-permeable AMPA receptors (Ca-A/KR). The Ca-A/KR channels transport Zn^2+^ and Ca^2+^ more easily than the NMDA receptor channels. Therefore, Ca^2+^ and Zn^2+^ toxicities are enhanced by the increased expression of Ca-A/KR channels. Zn^2+^ is also thought to be involved in the transcriptional regulation of Ca-A/KR channels because Ca-EDTA attenuates the ischaemia-induced downregulation of the *GluR2* gene [62]. These Zn^2+^-mediated neuronal cell death events can be explained by the Zn^2+^-mediated neurotoxicity hypothesis. Therefore, substances that prevent Zn^2+^-induced neuronal cell death are potential agents for VD and PD prevention or treatment. An investigation used various extracts collected from agriproducts, such as vegetables, fruits, and fish, and found that the Japanese eel (*Anguilla japonica*), mango fruit (*Mangifera indica* L.), and round herring (*Etrumeus teres*) had extracts that protected GT1-7 cells from Zn^2+^-induced neurotoxicity. The active fractions were separated from these extracts using high-performance liquid chromatography (HPLC), and the structures of their components were analysed by LC mass spectrometry. The active compounds included carnosine, citric acid, and histidine [25,32,73].

## 3. Carnosine Can Be a Therapeutic Agent for Cerebrovascular Dementia

### 3.1. Carnosine

Carnosine is a natural dipeptide composed of β-alanine and L-histidine (His). Carnosine and its analogues (anserine [1-methylcarnosine] and homocarnosine) are present in most vertebrate tissues, including those of birds; fish; and mammals, including humans [26,27]. In particular, carnosine is present in high levels in animals and fish that exercise frequently, such as horses, chickens, bonitos, and whales. In humans, carnosine levels have been reported to be higher in males, decrease with age, and be diet-dependent, with a vegetarian diet reducing carnosine levels in the skeletal muscle [74,75]. Similar to creatine and ATP, this dipeptide is also found in some muscles in amounts as much as 50–200 mM [76,77]. Carnosine levels in the muscle tissues of animals are adversely affected by factors such as trauma, shock, starvation, and injection. Infection and trauma may be associated with the dysregulation of cellular Ca and myocardial depression. Carnosine may be a regulator of cardiac cell contractility and [Ca^2+^]_i_ [78].

For example, intramuscular carnosine concentrations are 6–10 times higher in horses than in humans [27]. We analysed carnosine content in thoroughbred horse muscle and found that carnosine content was associated with a muscle fibre type [79]. Among the five equine muscle tissues (radius flexor, gill triceps brachii, masseter, gluteus medius, and sternocleidomastoid), the gluteus medius had the highest carnosine concentration. The glutaeus medius is enriched with type IIa (fast-twitch oxidative glycolytic muscle fibres) and IIx (fast-twitch glycolytic muscle fibre) fibres [80]. As these muscle fibres are primarily used during high-intensity exercise, carnosine may play an important role in high-intensity exercise. Carnosine may play a significant role in intracellular buffering due to its alkaline nature (pKa = 7.01) [81]. This buffering action is thought to play an important role in maintaining pH balance against the production of lactic acid, which causes muscle contraction fatigue due to acidosis during high-intensity anaerobic exercise and a decrease in intracellular pH. Therefore, the muscle carnosine concentration may be positively related to exercise performance [27,82]. Carnosine levels are higher in highly trained athletes than in untrained individuals [83]. Furthermore, the concentration of muscle carnosine can be increased by dietary supplementation with carnosine or β-alanine and delays fatigue during high-intensity exercise [83].

In addition, carnosine possesses various functions, such as anti-oxidation, anti-glycation, anti-crosslinking, and metal chelation, that mediate its beneficial effects in vivo [27]. Carnosine scavenges unpaired electrons containing both reactive oxygen and nitrogen and free radical scavenging and is involved in inhibiting lipid oxidation through free radical scavenging and metal chelation [84,85]. Moreover, the Maillard reaction—which produces many end-products, especially advanced glycation end-products, which contribute to the development of various senile diseases, such as AD, vascular sclerosis, atherosclerosis, and osteoarthritis—is inhibited by carnosine. In addition, carnosine exhibits anti-crosslinking properties that inhibit protein oligomerisation. N-acetylcarnosine is used as a treatment for cataracts because carnosine inhibits α-crystal fibrosis of the lens [86]. Polaprezinc, a Zn–carnosine complex, is effective in repairing gastrointestinal ulcers and other lesions [87]. Polaprezinc is also used in Zn supplementation therapy and shows protective effects against cadmium-induced lung injury [88]. 

### 3.2. Carnosine in the Brain

Carnosine and homocarnosine have been detected in the mammalian brain, but anserine has not yet been detected [89]. β-alanine is readily transported throughout the brain by Na^+^-dependent amino acid transport system(s) and acts as a neuromodulator/neurotransmitter, and it might, in theory, form carnosine [90]. Additionally, the carnosine transporter (peptide transporter 2) is expressed in some rat neuronal cells [91,92]. This suggests the putative ability of carnosine to cross the BBB. In the brain, carnosine is abundant in the olfactory bulb [93] and has been reported to be secreted from oligodendrocytes upon stimulation with glutamate [94]. Boldyrev et al. reported that carnosine is mainly present in the neurons or glial cells of the olfactory bulb, with levels in the olfactory bulb exceeding 1000 μmol/kg [27]. We developed a quantitative analysis method for carnosine and its analogues using an HPLC system equipped with a carbon column (Hypercarb™) [95] and investigated the distribution of these compounds in the rat brain [34]. The rat brain contains significant amounts of carnosine in the olfactory bulb but less carnosine in the cerebral cortex and cerebellum, and anserine was not contained in any region tested [34]. It has also been revealed that carnosine levels in the olfactory bulb increase from the foetal stage to maturity [96]. By contrast, homocarnosine levels show no change with postnatal age. These results are similar to those of previous studies described earlier. Biffo et al. showed that olfactory receptor neurons have abundant carnosine in their perikaria and cell processes, including the axonal projections to the main and accessory olfactory bulb [97]. Carnosine is rapidly synthesised and transported to the olfactory bulb via axonal transport [98]. In primary olfactory neurons, carnosine synthase activity is decreased by denervation and is restored by regeneration [99,100]. Carnosine in the olfactory bulb may be localised mainly in the sensory neurons [28]. 

Because carnosine forms complexes with Ca^2+^, Cu^2+^, and Zn^2+^ [101,102], it plays an important role in regulating Zn^2+^ homeostasis at synapses in neural tissues, especially in the carnosine- and Zn-rich olfactory lobes [27]. Disease-associated proteins (e.g., AβP, prion protein, and α-synuclein) are thought to be central to the pathogenesis of various neurodegenerative diseases known as ‘protein misfolding/proteinopathies/amyloid formation’, including AD, DLB, and PD. Carnosine prevents oxidation and glycation, both of which contribute to the crosslinking of proteins, and interferes with crosslinking and subsequent conformational changes [103]. The oligomerization of α-synuclein, which may play an important role in DLB and PD [104], is also inhibited by carnosine. Increasing evidence indicates that carnosine also inhibits AβP oligomerisation and neurotoxicity [105,106]. Corona et al. reported that the administration of carnosine inhibited AβP deposition and improved the learning ability of AD model mice [107]. Carnosine prevents oxidative stress and inflammation induced by AβP [108]. We previously reported that carnosine alleviates neuronal cell death by changing the conformation of the prion protein fragment peptide (PrP106-126) [109]. In addition, carnosine has been reported to reduce Mn-induced neurotoxicity [110]. These beneficial properties show that carnosine is thought to act as a ‘gatekeeper’ or ‘neuroprotectant’ in the brain [111]. 

### 3.3. Carnosine Suppresses Zn-Induced Neuronal Death

**Figure 4:**
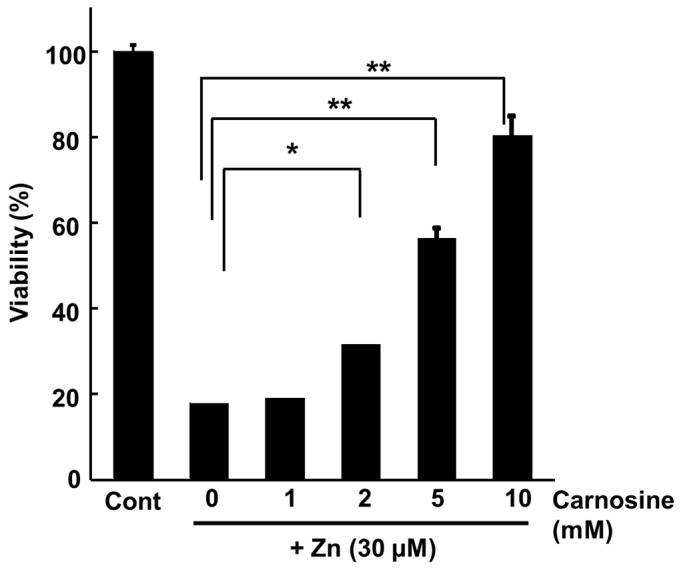
Protective activity against Zn^2+^-induced GT1-7 cell cytotoxicity. GT1-7 cells are treated with 30 μM of ZnCl_2_ with or without various carnosine levels. The viability is measured using the WST-1 assay 24 h after treatment. This figure was created using previously reported data [112]. The data are presented as means ± S.E.M., *n* = 6. * *p* < 0.01; ** *p* < 0.005.

**Figure 5:**
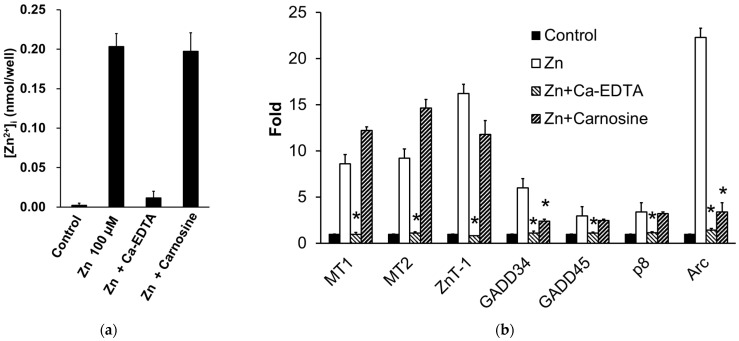
Effects of carnosine on the expression of Zn-induced factors. (**a**) Effect of carnosine on Zn influx into GT1-7 cells. GT1-7 cells are treated with 100 μM of ZnCl_2_ in the presence or absence of Ca-EDTA (0.2 mM) or carnosine (2.0 mM). After 30 min, [Zn^2+^]*_i_* was measured using a Metallo Assay Zinc LS kit (Metallogenics, Chiba, Japan) according to the manufacturer’s instructions. (**b**) Effects of carnosine on Zn-induced gene expression. GT1-7 cells are treated with 50 μM of ZnCl_2_ in the presence or absence of Ca-EDTA (0.5 mM) or carnosine (5.0 mM). After 6 h, the expression of metallothionein [*MT*]*1*, *MT2*, Zn transporter 1 (*ZnT-1*), growth arrest and DNA damage-induced gene 34 (*GADD34*), *GADD45*, *p8*, and a Ca^2+^-related gene (*Arc*) were analysed by RT-PCR, and the gene expression levels were normalised to β-actin. This figure was created using previously reported data [32,33]. Data are presented as the mean ± S.E.M., *n* = 3. * *p* < 0.01 versus the Zn group.

We found that carnosine has a protective effect on neurons against Zn^2+^-induced neurotoxicity (Figure 4) [112] and are investigating its mechanism. Our previous study showed that carnosine did not affect [Zn^2+^]_i_ or metal-related gene expressions, such as *MT1*, *MT2*, and *ZnT-1* (Figure 5a,b) [32,33]. Although carnosine can chelate Zn^2+^, it does not inhibit Zn^2+^ translocation by binding to extracellular Zn^2+^. By contrast, we found that carnosine inhibited the Zn^2+^-induced expression of ER-stress-related genes, such as *GADD34* and *CHOP*, and the Ca^2+^-related gene *Arc* (activity-related cytoskeletal protein) (Figure 5b) [33]. ER stress has been implicated in the development of various neurodegenerative diseases such as AD, PD, and ischaemia-induced neurodegeneration [32,113]. *GADD34*, a gene that encodes a sensor protein for ER stress, is induced by DNA damage and is thought to be involved in DNA repair and tumorigenesis [114]. *Arc* encodes a protein that resides in the dendrites and plays an important role in synaptic plasticity and memory consolidation. Increased neuronal activity in response to learning and brain-derived neurotrophic factors induces *Arc* expression [115]. Carnosine may protect neurons from Zn^2+^, not by inhibiting Zn^2+^ translocation, but by affecting ER stress and Arc-related pathways (Figure 3) because it attenuates neuronal cell death induced by ER stressors such as thapsigargin and tunicamycin [33]. Studies on experimental animals have suggested that carnosine protects against ischaemia-induced neurodegeneration in vivo [116,117,118,119]. Carnosine reduces 6-OHDA-induced neuronal cell death and inflammatory responses in GT1-7 cells through the marked inhibition of the 6-OHDA-induced upregulation of stress-related genes such as *Chop*, *GADD34*, and *Atf4*. Furthermore, it suppresses the 6-OHDA-induced activation of the SAPK/JNK signalling pathway by inhibiting ROS production [24]. In an early model of PD in rats using a single-site 6-OHDA injection into the striatum, pre-lesion administration of carnosine protected against 6-OHDA toxicity [120]. Therefore, carnosine may effectively prevent the onset and/or exacerbation of PD. 

### 3.4. Potential Uses of Carnosine and Its Derivative as Supplements

Dietary carnosine is absorbed into enterocytes of the small intestine via peptide transporter-1; from there, it is further transported to the lamina propria of the small intestine mucosa by peptide/histidine transporters 1 and 2. Limited amounts of carnosine are hydrolysed in intestinal cells [121], and nearly the entire amount enters the portal circulation [122]. In human blood, carnosine is widely believed to be rapidly degraded into β-alanine and histidine by circulating carnosinases (CN1). Both amino acids are reused for carnosine synthesis via ATP-dependent carnosine synthase (cytosolic enzyme) by the skeletal muscle, heart, and olfactory bulb [27,123,124,125]. In rat models, carnosine supplementation and β-alanine supplementation have been reported to increase the carnosine levels in the brain [126,127]. In humans, increasing one’s dietary intake of carnosine enhances its concentration in the skeletal muscle, brain, and heart [27]. Carnosine supplementation (40 mg/day) has been shown to be effective in treating patients with severe depressive disorder [128]. In addition, supplements containing anserine and carnosine (750 mg/250 mg per day) reportedly improve episodic memory in older adults [129] and mild cognitive impairment in individuals carrying the ε4 allele of apolipoprotein E, which is the most influential genetic risk factor for AD [130]. Therefore, dietary carnosine or related amino acids may be synthesised into carnosine in the brain and taken up into neuronal cells by oligopeptide transporters such as PEPT2, PHT1, and PHT2 [131]. A recent epidemiological study reported an inverse correlation between serum β-alanine levels and the aetiology of dementia [132]. Considering these factors and the fact that the carnosine levels in the body decrease with age [133], carnosine supplements may be beneficial for therapies for VD, AD, and other neurological disorders. Finding derivatives or analogues of carnosine that are resistant to degradation caused by CN1 is important for exploiting its full potential. Pharmacological variants of carnosine, such as carnosinol, a reduced carnosine derivative that is resistant to CN1, have been developed and show promise for use in the treatment of metabolic diseases, such as obesity and diabetes [134]. Additionally, balenine, found in marine mammals and reptiles, is a more stable natural analogue than carnosine in vivo and has potential uses as a dietary or ergogenic supplement [135].

## 4. Conclusions

Our hypothesis regarding the molecular pathways involved in Zn^2+^-induced neurotoxicity may aid in the development of preventive and therapeutic agents for VD and PD. Based on the activity of carnosine, we published two patents for carnosine and its related compounds (D-histidine) as drugs or supplements for the prevention and/or treatment of VD [136,137]. Carnosine has many beneficial properties, such as water solubility, heat inactivation, and non-toxicity, making it an excellent neuroprotective drug or supplement that benefits human health. Further studies are required to elucidate the molecular mechanisms by which carnosine prevents neurotoxicity.

## Data Availability

The data supporting the findings of this study are available from the corresponding author, Dai Mizuno, upon reasonable request.
